# GRAY MATTER CORRELATES OF ANXIETY, DEPRESSION, AND GAIT SPEED IN OLDER ADULTS WITH CHRONIC PAIN

**DOI:** 10.1093/geroni/igad104.3374

**Published:** 2023-12-21

**Authors:** Ania Lipat, David Clark, Yenisel Cruz-Almeida

**Affiliations:** The University of Florida, Gainesville, Florida, United States; The University of Florida, Gainesville, Florida, United States; The University of Florida, Gainesville, Florida, United States

## Abstract

Chronic pain and its comorbidities, (e.g., anxiety, depression, slow gait speeds) are a widespread, disabling condition prevalent among older adults. Worse anxiety and depression have been associated with slower gait speeds in individuals with chronic pain, and each comorbidity has been associated with structural gray matter changes; however, results are inconclusive or limited. Further investigation into these relationships may provide insight into neural mechanisms through which anxiety and depression contribute to slow gait speeds in this population. Therefore, the aim of this study was to identify gray matter measures associated with anxiety, depression, and gait speed in older adults with chronic pain. Participants (n = 31) walked across a GAITRite mat to obtain gait speed, and completed the State-Trait Anxiety Inventory (STAI), Center for Epidemiologic Studies Depression Scale (CES-D), and a T1-weighted anatomical scan. STAI score (mean = 7, β = - 0.015, p = 0.010) and CES-D score (mean = 30, β = - 0.014, p = 0.016) were significantly associated with gait speed. CES-D score was associated with left cuneus gyrus (β = 0.017, p = 0.0002) and frontal inferior sulcus surface area (β = 0.019, p = 0.002). Gait speed was associated with right inferior temporal gyrus surface area (β = 0.436, p = 0.019). STAI score was not associated with any measure. In conclusion, anxiety and depression symptoms contribute to gait slowing in older adults with chronic pain but without clinical depression or anxiety. This relationship, however, is not reflected in each comorbidity’s structural neural correlates.

